# Structural valve deterioration of a mitral Carpentier-Edwards pericardial bioprosthesis in an 87-year-old woman 16 years after its implantation

**DOI:** 10.1186/1749-8090-6-88

**Published:** 2011-07-05

**Authors:** Hiroshi Ito, Kensuke Sakata, Takashi Haruki, Yurio Kobayashi

**Affiliations:** 1 Department of Cardiovascular Surgery, Saiseikai Shimonoseki General Hospital, 8-5-1 Yasuoka, Shimonoseki, 759-6603, Yamaguchi

## Abstract

The second-generation pericardial valve, the Carpentier-Edwards perimount bioprosthetic (CEP) valve, shows dramatically improved durability as compared to the first-generation pericardial valve, and excellent performance has been obtained, in both the aortic and mitral positions. Especially in elderly patients with an implanted CEP valve, reoperation due to structural valve deterioration (SVD) is rarely required. Here, we report the case of an 87-year-old woman with an explanted CEP valve in the mitral position due to SVD, 16 years after its implantation.

## 

An 87-year-old woman was admitted to our hospital with acute heart failure, NYHA class IV. An echocardiography revealed severe mitral regurgitation and heart failure with pulmonary hypertension. She had been diagnosed as having severe mitral stenosis and had undergone mitral valve replacement with a 27-mm Carpentier-Edwards mitral pericardial valve (model 6900) 16 years prior (at 71 years old) to the present admission. An echocardiography performed 3 months prior to this admission revealed mild mitral stenosis and regurgitation; however, there were no associated clinical symptoms. Prior to the present admission, she was brought to the hospital with dyspnea of acute onset. A transesophageal echocardiogram revealed severe mitral regurgitation due to structural valve deterioration (SVD) of the implanted CEP valve, moderate TR, and severe pulmonary hypertension, with a PAP of 93 mm Hg (Figure 1). She was initially treated with furosemide and cariperitide, which produced slight improvement of the heart failure; however, reoperation was found to be necessary. The reoperation was performed 9 days after admission via a median sternotomy and under moderate hypothermic cardiopulmonary bypass with antegrade cold crystalloid cardioplegic arrest. The mitral valve was examined through an incision in the left atrium. A tear was noted in one of the leaflets of the implanted CEP mitral valve, which was thought to be the cause of the severe mitral regurgitation. The cuff of the valve was covered with thick intima; however, the leaflets were relatively soft. The valve was resected, and a 27-mm Mosaic mitral bioprosthesis was implanted in its place. Tricuspid valve ring annuloplasty was performed with a 30-mm MC3. The patient was extubated on the day after the surgery and discharged from our hospital on day 20 after the operation.

**Figure 1:**
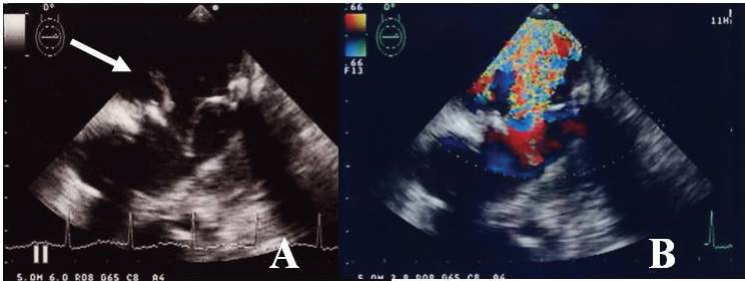
**Echocardiography revealing prolapse of the Carpentier-Edwards Perimount bioprosthesis (white arrow) (A) and severe mitral regurgitation (B)**.

The macroscopic findings of the deteriorated valve were as follows (Figure 2A, B): The stenosis of the valve was caused by the host tissue overgrowth restricting the mobility of the leaflets. A tear was evident in leaflet 1 at commissure 2, which measured approximately 14 mm, beginning from the commissure, along the ring of the prosthetic valve.

**Figure 2:**
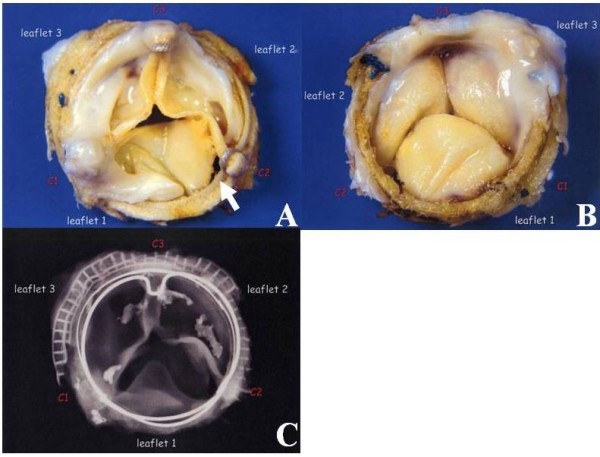
**Explanted Carpentier-Edwards Perimount bioprosthesis** (view from the left ventricule: A, view from the left atrium: B showing a tear in leaflet 1 (white arrow). X-ray of the valve showed calcification on leaflets 2 and 3, but not on leaflet 1 (C).

Calcification was detected in the x-ray on leaflets 2 and 3, which were covered by a dense layer of host tissue overgrowth (Figure 2C).

## Discussion

Marchand et al. reported an actuarial freedom rate from structural valve deterioration (SVD) in patients receiving implantation of the CEP valve, 6900 model, in the mitral position of 59.2%±6.6% in patients under 60 years of age, 76.0%±6.3% in patients between 60 and 70 years of age, and 100% in patients over 70 years of age [1]. A literature search to the best of our ability revealed no cases that had undergone CEP valve implantation in the mitral position at more than 70 years of age, with subsequent SVD and explantation of the valve. The average life expectancy of Japanese is quite long, being 79.59 years in males and 86.44 years in females, and the average expected length of life at 70 years is 15.1 years in males and 19.61 years in females (Japan Ministry of Health and Welfare 2009 http://www.mhlw.go.jp/toukei/saikin/hw/life/life09/). Our patient had undergone her first implantation of a mitral bioprosthetic valve at the age of 71 years; her postoperative course had been excellent, and her condition had remained satisfactory for more than 15 years without warfarin. Unfortunately, she developed SVD suddenly, 16 years after the valve implantation, and needed a reoperation at the age of 87 years. However, she was healthy enough even at this age to tolerate heart surgery.

Bioprosthetic valve implantation in the mitral position is usually performed in patients who are more than 60 or 70 years old. As reported here, especially in Japanese subjects who have a long life expectancy, SVD of an implanted valve at over 70 years of age may occur, possibly necessitating reoperation. Notwithstanding, bioprosthetic valves must be selected for elderly patients, considering the risk of thromboembolism, and consequently of hemorrhage associated with the use of warfarin, in patients with a mechanical valve. Cannegier et al. reported that the risk of hemorrhage in patients over 70 years of age with an implanted mechanical valve was 5.6%/pt-year, which is twice as high as the risk reported in patients who are less than 70 years old [2]. Holper et al. reported that the actuarial freedom rate from major bleeding at 15 years was 88%±4% in patients with an implanted bioprosthetic valve, and 57%±1.1% in those with an implanted mechanical valve [3]. In agreement with this, Marchand et al. reported that the actuarial freedom rate from major bleeding in patients with an implanted CEP valve was 86.6%±3.2% at 14 years [1]. These data suggest that a bioprosthetic valve is superior to a mechanical valve from the viewpoint of the risk of major bleeding. As reoperation can also be performed safely in elderly patients at present, it might be better to select a bioprosthetic valve for elderly patients, notwithstanding the risk of reoperation due to SVD more than 10 years later [4].

In regard to the pattern of SVD of a CEP valve, calcification (70.4%-73%), valve tear (18.5%-20%), or and both (7%-11.1%) have been reported [1,5]. The main cause of SVD in our present patient was a tear of one of the valve leaflets, which probably occurred suddenly, causing severe mitral regurgitation and heat failure. The torn leaflet showed no calcification on a plain radiograph, while the other two leaflets showed calcification. The differential calcification of the leaflets of the same CEP valve is thought to be related to the different bovine origin of the component tissues of the valve. The imbalance of calcification in the three leaflets can cause imbalance of the tension between these leaflets, increasing the risk of leaflet tear [6,7]. In our patient, the tear was noted in the leaflet that showed no calcification, while the other two leaflets showed calcification. An interesting report on the Mosaic valve, which is of single porcine origin, indicates excellent durability of the valve in the mitral position, with no evidence of SVD at 10 years [8]. If this is true, the different pattern of SVD of the CEP valve, especially the occurrence of the tear, may be attributable to its different origin. On the other hand, the quality of each component could also be different even in the single porcine valve, hence further investigation is necessary.

In conclusion, in Japanese patients with a high life expectancy, SVD of an implanted bioprosthetic valve can occur in patients undergoing the valve surgery even after 70 years of age. While it would be desirable to implant bioprosthetic valves for elderly patients to avoid the risk of major bleeding, reoperation may become necessary in patients living long after the surgery. We have described the first case of a patient who developed SVD and explantation of a CEP valve, 16 years after it was implanted, in a patient who was over 70 years of age at the time of the surgery.

## Competing interests

The authors declare that they have no competing interests.

## Authors' contributions

HI performed the procedure. KS, TH, and YK participated in the procedure. All authors read and approved the final manuscript.
